# Quantum Dot Nanobeads Based Fluorescence Immunoassay for the Quantitative Detection of Sulfamethazine in Chicken and Milk

**DOI:** 10.3390/s21196604

**Published:** 2021-10-03

**Authors:** Daixian Wei, Jintao Liu, Zexiang Wang, Shu Zhou, Suhua Wang, Weipeng Tong, Juan Peng

**Affiliations:** 1 State Key Laboratory of Food Science and Technology, Nanchang University, Nanchang 330047, China; 15395044739@163.com (D.W.); L13629131177@163.com (J.L.); wangzexiangky2020@163.com (Z.W.); bailizhousu@163.com (S.Z.); W18720072745@163.com (S.W.); cuvbvdx@163.com (W.T.); 2 Jiangxi-OAI Joint Research Institute, Nanchang University, Nanchang 330047, China; 3 School of Food Science and Technology, Nanchang University, Nanchang 330047, China

**Keywords:** lateral flow immunoassay (LFIA), quantum dot nanobeads (QBs), class-specific monoclonal antibody, sulfamethazine (SMZ), chicken

## Abstract

Sulfamethazine (SMZ) as a broad antibiotic is widely used in livestock and poultry. However, the abuse of SMZ in livestock feed can lead to SMZ residues in food and the resistance of bacteria to drugs. Thus, a method for the detection of SMZ in food is urgently needed. In this study, quantum dot (QD) nanobeads (QBs) were synthesized by encapsulating CdSe/ZnS QDs using a microemulsion technique. The prepared QBs as signal probes were applied in lateral flow immunoassay (LFIA) for the detection of SMZ in chicken and milk. Our proposed method had limits of detection of 0.1138–0.0955 ng/mL and corresponding linear ranges of 0.2–12.5, 0.1–15 ng/mL in chicken and milk samples, respectively. The recovery of LFIA for the detection of SMZ was 80.9–109.4% and 84–101.6% in chicken and milk samples, respectively. Overall, the developed QBs-LFIA had high reliability and excellent potential for rapid and sensitive screening of SMZ in food.

## 1. Introduction

Sulfanilamides (SAs) had been used as a family of broad-spectrum synthetic bacteriostatic antibiotics given to food animals for prophylactic or therapeutic purposes after G. Domagk found that SAs can inhibit bacterial growth [1,2]. Meanwhile, SAs are chemotherapeutic agents widely used as antibacterial drugs in human pharmaceuticals and veterinary practice [3].

SMZ is a derivative of SAs and widely used in livestock [4] and poultry breeding. However, abuse in livestock feed can cause SMZ residues in food [5,6,7]. Widespread use of antibiotics can increase bacterial resistance to drugs; therefore, excessive use of antibiotics will pose a threat to human safety [8]. SMZ exposed in the environment can enter the human body through the food chain and pose a serious threat to human health [9]. Therefore, an effective system that can detect the residues of SMZ needs to be developed. The maximum residue limit of SAs in animal-derived food has been established at 100 μg/kg [10,11].

Many sensitive and specific methods can be used to detect SMZ, including high-performance liquid chromatography [12,13], gas chromatography-mass spectrometry [14], and waveguide interrogated optical immunosensor [15]. Although these methods are relatively sensitive and specific, they require a complex sample preparation process and trained personnel and expensive equipment to perform the operations [16]. LFIA is based on specific recognition of antigens and antibodies. LFIA can be considered a convenient and practical test, and it represents a classic transformation between sample-to-laboratory so that it can be easily used to make decisions [17]. Compared with other methods, it is an analytical method with many superior advantages, such as a short analysis time, long-term stability of test strips, ease of use, and cost-effectiveness [18,19,20,21,22]. Given these advantages, some nanoparticle-based LFIAs have been widely used in qualitative and quantitative detection [23,24]. For example, conventional LFIA using colloidal gold as a label exhibits relatively low sensitivity because of its insufficient brightness. Shim et al. established a rapid immunochromatographic (ICG) strip based on a conjugate of colloidal gold and monoclonal antibody (mAb) for the detection of SMZ in meat and egg samples. The detection limit of the ICG strip is 2 ng/mL [25]. O’Keeffe et al. used mouse anti-rat IgG F(abV)2 fragment-specific antibody, adsorbed to colloidal carbon, as the detection ligand in the LFIA. The LFIA device had a cutoff value of 6.3 ng/mL in diluted (1/10) urine [26]. Compared with traditional methods, polymer nanocontainers have attracted increasing interest in signal amplification systems. The fluorescence-based immunoassay is widely used in biotechnology and clinical testing. Antibodies or antigenic proteins can be easily labeled with a variety of fluorophores for fluorescence detection [27]. For improving the sensitivity of traditional LFIA, the use of QBs has recently attracted our attention due to their incomparable optical properties, such as broad UV excitation with narrow fluorescent emission spectra, large molar extinction coefficient, and high quantum yield [28,29].

In the present work, a novel LFIA was established based on QBs as the label for the sensitive detection of SMZ in chicken and milk. Through the assay mentioned above, QBs-LFIA may be a remarkable method for the sensitive detection of other targets at low concentrations to ensure food safety.

## 2. Materials and Methods

### 2.1. Reagents and Materials

SMZ, sulfaguanidine, sulfanilamide, sulfamonomethoxine, sulfadiazine, sulfamethoxazole, sulfacetamide, sulfathiazole, sulfamethizole, and other non-SA antibiotics (lomefloxacin, amantadine, florfenicol) were obtained from J&K Scientific Ltd. (Shanghai, China), and the stock solution of these SAs in methanol (5 mg/mL) was prepared for further use. Hydrophobic octadecylamine-coated CdSe/ZnS QDs were obtained from Ocean NanoTech, LLC (San Diego, CA, USA). Bovine serum albumin (BSA), poly (maleicanhydride-alt-1-octadecene) (PMAO), and goat anti-mouse antibody was obtained from Sigma (St. Louis, MO, USA). Poly (methyl methacrylate) (PMMA), 1-ethyl-3-(3-dimethylaminopropyl) carbodiimide (EDC), and sodium dodecyl sulfonate (SDS) were purchased from Aladdin Bio-Chem Technology Co., Ltd. The monoclonal antibody (mAb) against SMZ was provided by Wuxi Zodoboer Biotech. Co., Ltd. (Wuxi, China). The detective antigen SMZ − BSA was prepared in our laboratory. All of the solvents and other chemicals were of analytical reagent grade. Nitrocellulose (NC) membrane was supplied by Millipore (Bedford, MA, USA). A polyvinylchloride backing pad, an absorbent pad, a sample pad, and a conjugate pad were purchased from Shanghai Kinbio Tech. Co., Ltd. (Wuxi, China). The BioDot XYZ platform, which was equipped with an automated motion control, was acquired from BioDot (Irvine, CA, USA). Fluorescence intensity strip reader for the sake of recording the signal of QBs-LFIA was purchased from Hangzhou Hemai Technology Co., Ltd. (Hangzhou, China).

### 2.2. Preparation of QBs

The QBs were obtained by encapsulating CdSe/ZnS QDs using the microemulsion technique [30,31]. CdSe/ZnS QDs CHCl_3_ stock solution (40 mg/mL) was added into PMMA/PMAO CHCl_3_ stock solution (PMMA: 60 mg/mL; PMAO: 40 mg/mL). Then, the solution was added into SDS aqueous solution by using an ultrasonic homogenizer for 3 min in an ice bath. Subsequently, the CHCl_3_ was removed by using a rotary evaporator. The mixture was centrifuged at 13,000 r/min at 4 °C for 15 min and washed three times with distilled water. The obtained QBs were purified via centrifugation and then resuspended in 1 mL of 0.01 M NaOH solution for 8 h to modify the carboxyl on the surface of the QBs. Finally, the carboxylated QBs were washed three times with Millipore water.

### 2.3. Preparation and Characterization of QBs-mAb

The anti-SMZ monoclonal antibodies were directly bound to QBs. An amount of 5 μL of EDC (0.5 mg/mL), 5 μL of QBs (12.6 mg/mL), and 50 μL mAb (0.2 mg/mL) was added to 0.5 mL of 0.01 M PB buffer (pH7.0) with gentle stirring for 1 h at room temperature. Then, 0.1 mL BSA (10% wt/vol) was added to block the remaining sites for 1 h. The resulting mixture was centrifuged at 13,500 r/min for 10 min. Then the QBs and the anti-SMZ mAb conjugates (QBs-mAb) were resuspended in 0.1 mL of PBS (0.01 M, pH 7.4). The hydration size of the free QBs and QBs-mAb probes were given analysis by using a particle size analyzer (Malvern Instruments Ltd., Worcestershire, UK).

### 2.4. Assembling of QBs-LFIA Sensor

The goat anti-mouse IgG and SMZ − BSA were diluted with PBS (0.01 M), and then the two mixtures were sprayed to control lines (C) and test lines (T) on the nitrocellulose membrane and dried at 37 °C overnight. The distance between the T and C lines was about 5 mm. After drying at 60 °C for 2 h, the sample pad, NC membrane, and absorption pad were neatly attached to the plastic pad, cut into 4 mm wide strips, placed in a plastic box, and stored in a drying box at room temperature.

### 2.5. Procedure of QBs-LFIA

**Scheme 1:**
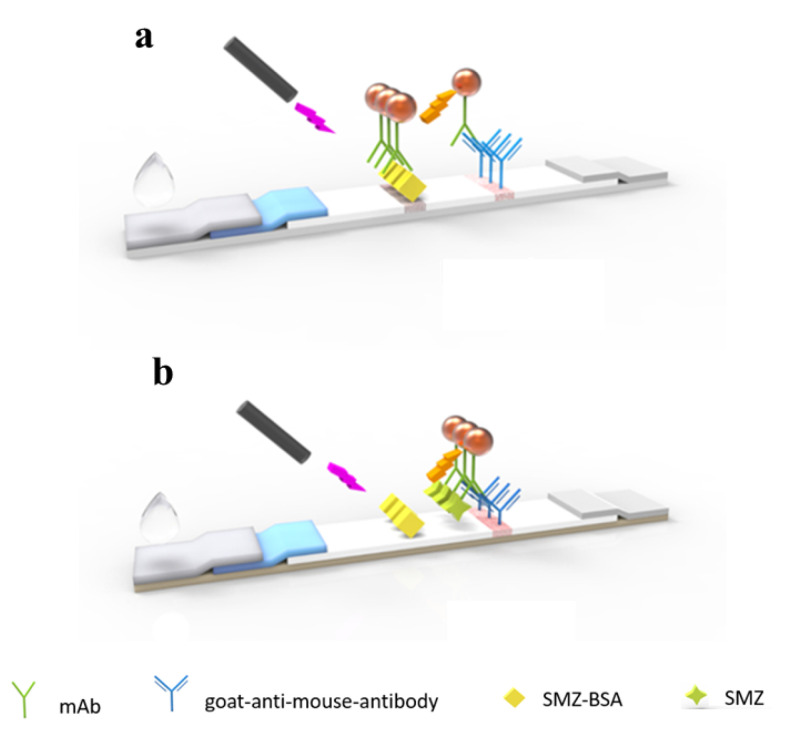
Schematic illustration of the QBs-LFIA for SMZ detection. (**a**) Negative, (**b**) positive.

SMZ stock solution was diluted in 0.01 M PBS, 0.1 mL of sample solution, and 2 μL of QBs-mAb probe, and was mixed for 5 min and then added on the sample pad of the LFIA test strip. After 20 min of reaction, the fluorescence intensity on the test line was recorded by the corresponding reader. As shown in Scheme 1, with the help of the absorption pad, the conjugates migrate to the NC membrane. When the sample is negative, the QBs-mAb probe binds to the antigen on the T line. By contrast, when SMZ is present in the sample, the QBs-mAb probe combines with SMZ preferentially, and the excess probe captures the antigen on the T line. The more SMZ in the sample, the lower the fluorescence intensity of the T line. All the QBs-mAb probes should react with the goat anti-mouse IgG to form a fluorescent band on the C line.

### 2.6. Optimization of the Key Parameters

For the best performance of our proposed QBs-LFIA, we used single-factor analysis to optimize the key factors that could influence the limit of detection, such as the coupling pH, concentration of EDC, amount of mAb decorated to QBs, concentration of SMZ − BSA on the T line, amount of QBs-mAb, and immunological kinetic reaction time [32].

### 2.7. Study on Immune Dynamic Response

The immune response of antigen antibody on the immunochromatographic strip is a dynamic process in which the T-line signal intensity changes dynamically with the increase of the reaction time. The signal strength will rise slowly and then gradually stabilize. Therefore, in order to make the experimental results more reliable, immunodynamic analysis was performed. After incubating 100 μL of negative PBS (without SMZ) and positive PBS (with 1 ng/mL SMZ) with 5 μL of QB-labeled antibody for 5 min, they were dropped into the sample hole on the test strip. After 2 min, the signal strength of the T-line was measured with a reader every 1 min, and the detection continued for 50 min. Using the detection time as the abscissa and the T-line fluorescence intensity and inhibition rate as the ordinate, the immune response dynamic curve was drawn to select the optimal detection time.

### 2.8. Establishment of Standard Calibration Curve in PBS

SMZ (0, 0.01, 0.05, 0.1, 0.25, 0.5, 1.0, 2, 2.5, 5.0, 10.0, 20, 25, 50, 100 ng/mL) was added to PBS. A standard curve was established using the logarithm of the concentration of SMZ and fluorescence intensity of the T line. The limit of detection (LOD) was defined as IC_90_ [15]. All experiments were performed in triplicate.

### 2.9. Specificity Assessment

The specificity of QBs-LFIA was tested at a concentration of 20.0 ng mL^−1^ with structurally similar analogs, including SMZ, sulfaguanidine, sulfapyridine, sulfanilamide, sulfamonomethoxine, sulfadiazine, sulfamethoxazole, sulfacetamide, sulfathiazole, sulfamethizole, and other non-SAs antibiotics (lomefloxacin, amantadine, florfenicol). All experiments were performed in triplicate.

### 2.10. Sample Preparation

#### 2.10.1. Chicken Sample Pretreatment

Samples of chicken were smashed and stored at −20 °C for further analysis. An amount of 10 µL of working standard solution was added to the chicken sample (5.0 g) in a centrifuge tube, and the mixture was vortexed for 30 s. After being sonicated for 30 min and centrifuged at 8500 rpm at 4 °C for 15 min, the resulting supernatant solution was concentrated to near dryness under nitrogen at 50 °C. The resulting residue was redissolved in 1 mL PBS for further analysis.

#### 2.10.2. Milk Sample Pretreatment

For sample treatment, 5 g milk sample was centrifuged at 9000 rpm for 20 min. The supernatant solution was collected and diluted 10-fold for further analysis.

### 2.11. Detection of SMZ in Real Samples

#### 2.11.1. Establishment of Standard Calibration Curve in Real Samples

SMZ (0, 0.01, 0.05, 0.1, 0.25, 0.5, 1.0, 2, 2.5, 5.0, 10.0, 20, 25, 50, 100 ng/mL) was spiked to the chicken and milk. We established a standard curve using the logarithm of the concentration of SMZ and fluorescence intensity. All experiments were performed in triplicate.

#### 2.11.2. Recovery of SMZ on the QBs-LFIA System in 2 Food Matrices

SMZ (0.5, 5, and 7.5 ng/mL) was added to the 2 food matrices. The recovery and coefficient of variation (CV) of SMZ in the real samples were quantitatively analyzed on the LFIA system. All experiments were performed in triplicate.

#### 2.11.3. Analysis of HPLC

Eight chicken samples and eight milk samples were selected and determined by the developed QBs-LFIA and HPLC [33] to verify the reliability of the QBs-LFIA.

## 3. Results and Discussion

### 3.1. Characterization of QBs and QBs-mAb

**Figure 1:**
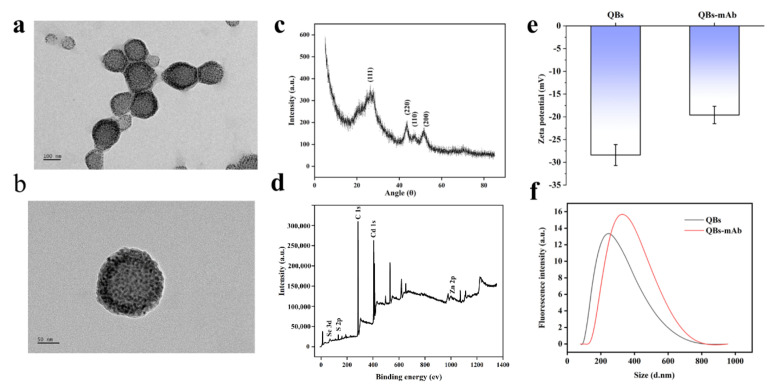
Characterization of QBs and QBs-mAb. (**a**) High-resolution TEM image of the QBs. (**b**) Individual QB at high magnification. (**c**) XRD patterns of QBs, (**d**) XPS survey of QBs, (**e**) zeta potential of QBs before and after coupling with mAb. (**f**) Size distribution of the hydrodynamic diameter of QBs and QBs-mAb.

Figure 1a shows a transmission electron microscope (TEM) image of the QBs. The image indicates that the QBs have relatively uniform particle size distribution and present regular quasi-spherical shapes with an average diameter of 158 nm, while Figure 1b shows an individual QB at high magnification. XRD patterns in Figure 1c show that the three distinct QBs peaks positioned at 26.6, 43.5, and 51, which were similar to the QDs in a previous report, still retained CdSe/ZnS QDs structural characteristics [34,35]. X-ray photoelectron spectroscopy was employed to demonstrate the chemical composition and surface chemical states of QBs. As Figure 1d shows, the survey spectra of QBs were composed of Zn, Cd, and Se elements, which is consistent with the QDs spectra, as a previous work reported [36,37]. In Figure 1f, DLS analysis indicates that the hydrodynamic diameter of the QBs is about 255 nm, while that of the QBs-mAb raises it to 315 nm. Meanwhile, the zeta-potential of QBs and QBs-mAb was monitored; as Figure 1e shows, the zeta potential of QBs is −28 mV, while the QBs-mAb raises it to −18 mV. The results demonstrate that the anti-SMZ mAb was favorably coupled on the surface of the QBs.

### 3.2. Optimization of QBs-LFIA

#### 3.2.1. Optimization of the Coupling pH

**Figure 2:**
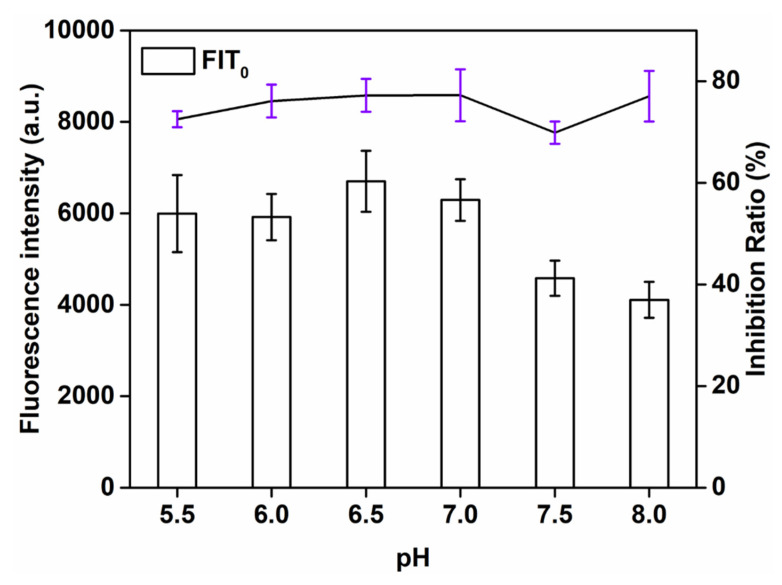
Optimization detection conditions of QBs-LFIA. Effects of pH value.

It is necessary to optimize the pH of the label because it affects the activity of the antibody and the conjugation between the antibody with the QBs. We evaluated the effect of pH value through analyzing the fluorescence intensity, which varied, as the pH values ranged from 5.5 to 8.0. As can be seen from Figure 2, when the QBs-labeled antibody is sufficient, the detection signal strength of the T line fluctuates according to the coupling pH. The T-line signal reached its maximum strength at pH = 6.5, but the inhibition rate was lower than that at pH = 7.0. Both high FIT_0_ and inhibition ratio were attained at pH 7.0. Therefore, pH = 7.0 was chosen as the optimal pH.

#### 3.2.2. Optimization of the Concentration of EDC

**Figure 3:**
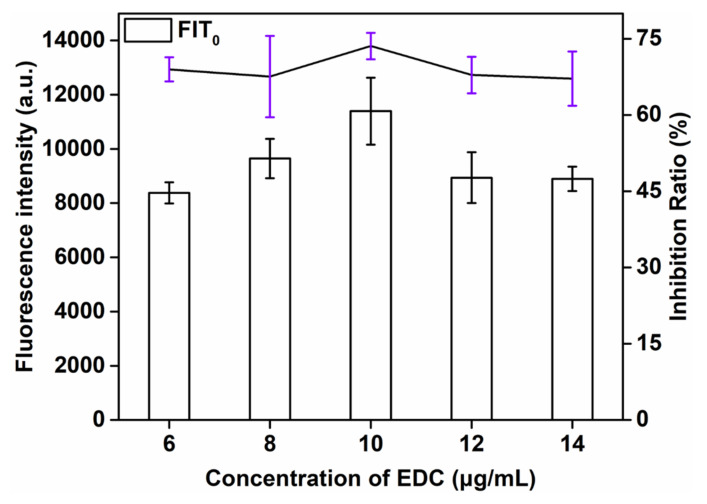
Optimization detection conditions of QBs-LFIA. Effects of concentration of EDC.

The effect of the concentration of EDC, an important factor influencing the F of the biosensor, on the sensitivity of QBs-LFIA was also investigated. The successful preparation of the probe was mainly attributed to covalently coupling the amino group of anti-SMZ mAb with the carboxyl group of QBs in the presence of EDC. This study explored the influence of the concentration of EDC (6, 8, 10, 12, and 14 μg/mL) on the detection signal strength and inhibition rate. As can be seen from Figure 3, the signal strength and inhibition rate both increased with the additive amount of EDC consumption and reached the maximum value at 10 μg/mL (73.8%). When the concentration of EDC continued to increase, the signal strength and inhibition rate showed a downward trend. Therefore, the optimal amount of EDC was 10 μg/mL.

#### 3.2.3. Optimization of the Amount of mAb

**Figure 4:**
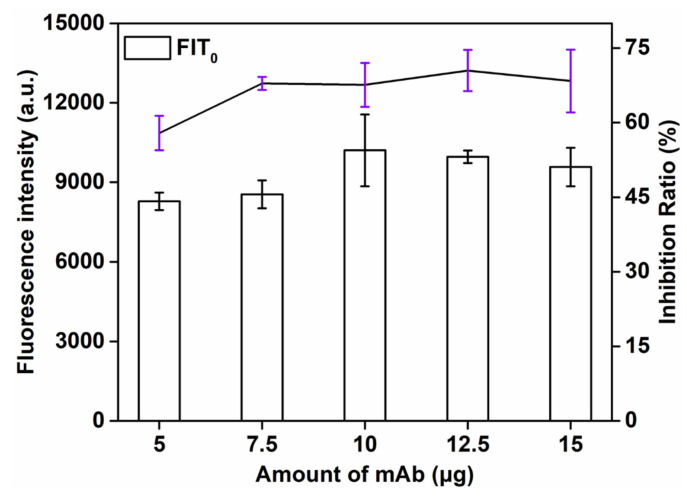
Optimization detection conditions of QBs-LFIA. Effects of amount of mAb.

This study explored the influence of the amount of labeled antibody (5, 7.5, 10, 12.5, 15 μg) on the detection signal strength and inhibition rate. As can be seen from Figure 4, the signal strength increased with the increase in the amount of labeled antibody. The higher the volume of the antibody, the more SMZ − BSA binding and the stronger the T-line signal. When 12.5 μg of mAb was applied, the maximum inhibition rate (72.3%) was obtained. However, the T-line signal was the strongest when the amount of mAb was 10 μg, and the inhibition rate (70.8%) was a little lower than the amount of mAb at 12.5 μg. Finally, the optimal amount of mAb was 12.5 μg.

#### 3.2.4. Optimization of the Concentration of SMZ − BSA on the T Line

**Figure 5:**
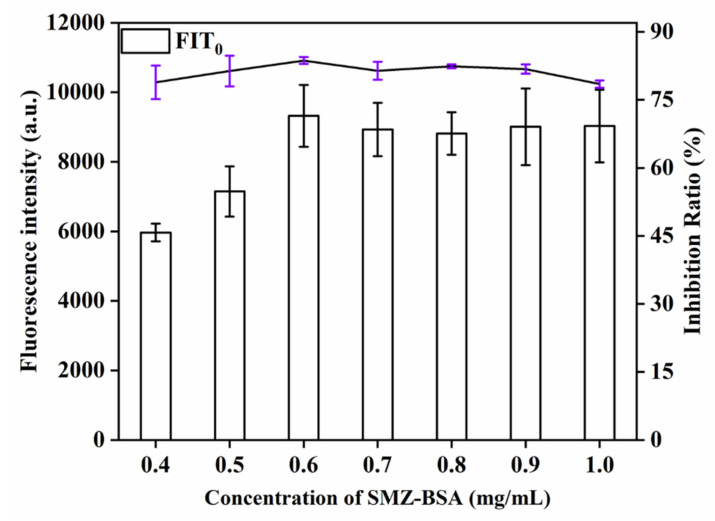
Optimization detection conditions of QBs-LFIA. Effects of concentration of SMZ − BSA.

This study explored the influence of different concentrations (0.4, 0.5, 0.6, 0.7, 0.8, 0.9, 1.0 mg/mL) of SMZ − BSA on detection signal strength and inhibition rate. As can be seen from Figure 5, when the QBs-labeled antibody was sufficient, the detection signal strength of the T line increased with the increase of SMZ − BSA concentration. With increasing amount of SMZ − BSA, more QBs antibody conjugates can be captured, and the T line will show a stronger signal. The T-line signal was the strongest and the inhibition rate (81.2%) was the highest when the concentration of SMZ − BSA was 0.6 mg/mL; when the concentration of SMZ − BSA continued to increase, the signal strength and inhibition rate decreased. Therefore, 0.6 mg/mL was chosen as the optimal concentration of SMZ − BSA on the T line.

#### 3.2.5. Optimization of the Amount of QBs-mAbs Probe

**Figure 6:**
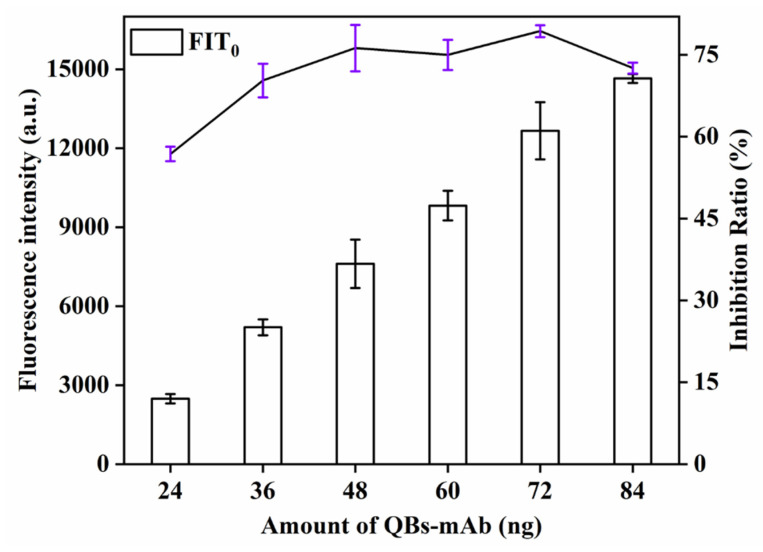
Optimization detection conditions of QBs-LFIA. Effects of amount of QBs-mAb.

The effect of the amount of QBs-mAbs probe, an important factor influencing the F of the biosensor, on the sensitivity of QBs-LFIA was also investigated. This study explored the influence of the amount of QBs-mAbs probe (24, 36, 48, 60, 72, 84 μg) on detection signal strength and inhibition rate. As can be seen from Figure 6, the signal strength and inhibition rate (77.8%) increased with increasing amount of QBs-mAbs probe and reached the maximum value at 72 μg. When the amount of QBs-mAbs probe continued to increase, the signal strength and inhibition rate showed a downward trend. Therefore, the optimal amount of EDC was 72 μg.

#### 3.2.6. Immune Dynamic Response Analysis of QBs-LFIA

**Figure 7:**
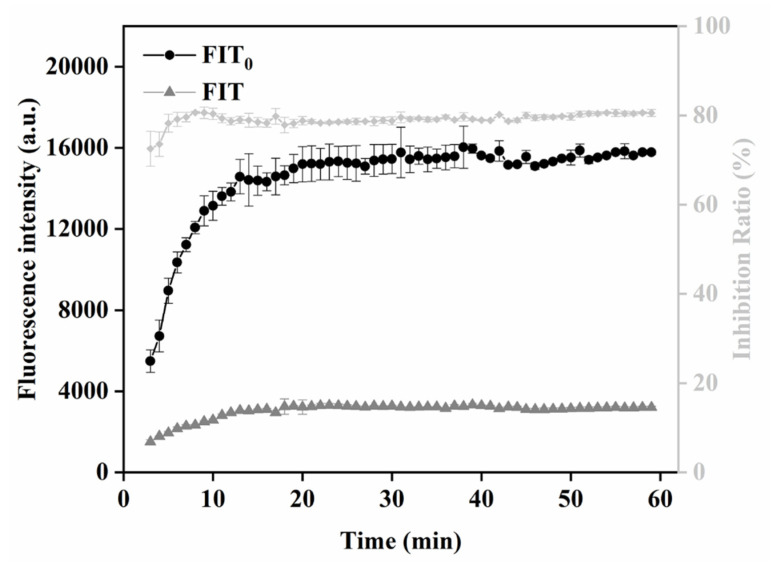
Optimization detection conditions of QBs-LFIA. Immunoreaction dynamic of FIT of negative (0 ng/mL) and positive (5 ng/mL) samples on the fluorescence intensity of T line and inhibition ratios.

In this study, the relationship between T-line signal and immune response time was monitored by recording the fluorescence intensity of the T line and C line every 1 min. FIT_0_ and FIT represent the change value of fluorescence intensity along the T line of negative and positive sample strips with time, respectively. The control negative and positive samples were PBS containing QBs-mAb and PBS containing SMZ and QBs-mAb, respectively. As can be seen from the trend in Figure 7, FIT_0_, FIT, and inhibition rate tend to be stable when the sample adding time exceeds 20 min. Therefore, 20 min was selected as the optimal detection time.

#### 3.2.7. Evaluation of QBs-LFIA

##### Determination of Detection Limit and Linear Range

**Figure 8:**
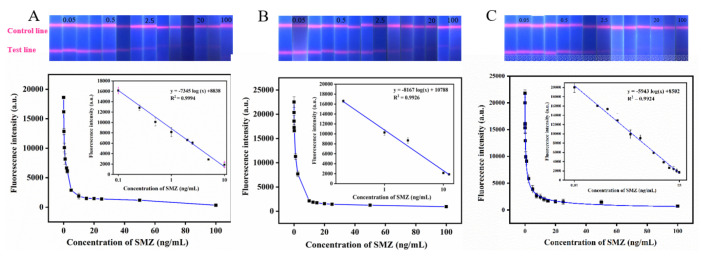
The digital photos under UV light of the QBs-LFIA test strips and corresponding standard calibration curves for SMZ were constructed by plotting the fluorescence intensity against the logarithm of different concentrations of SMZ in (**A**) PBS, (**B**) chicken, and (**C**) milk samples.

Under the optimal process parameters, the QBs-based immunochromatographic method established in this study was used to detect PBS solutions with different SMZ concentrations. The standard curve was drawn using the fluorescence intensity of the T line as the ordinate and the logarithm of the corresponding standard concentration as the abscissa. As shown in Figure 8A, the intensity gradually decreases with the increase of SMZ concentration. The equation of the standard curve is y = −7345 log (x) + 8838 (R^2^ = 0.9994), where y represents the fluorescence intensity, and x represents the logarithm value of SMZ concentration. By calculation, the LOD of SMZ detection in PBS buffer by QBs-based immunochromatography was 0.1074 ng/mL, and the corresponding linear detection range was 0.1–10 ng/mL. Notably, Table 1 also compares the LOD and the linear range of the QBs-LFIA in this study with others, and results indicate that QBs-LFIA have advantages in linear range and LOD.

##### Specificity of the QBs-LFIA

**Figure 9:**
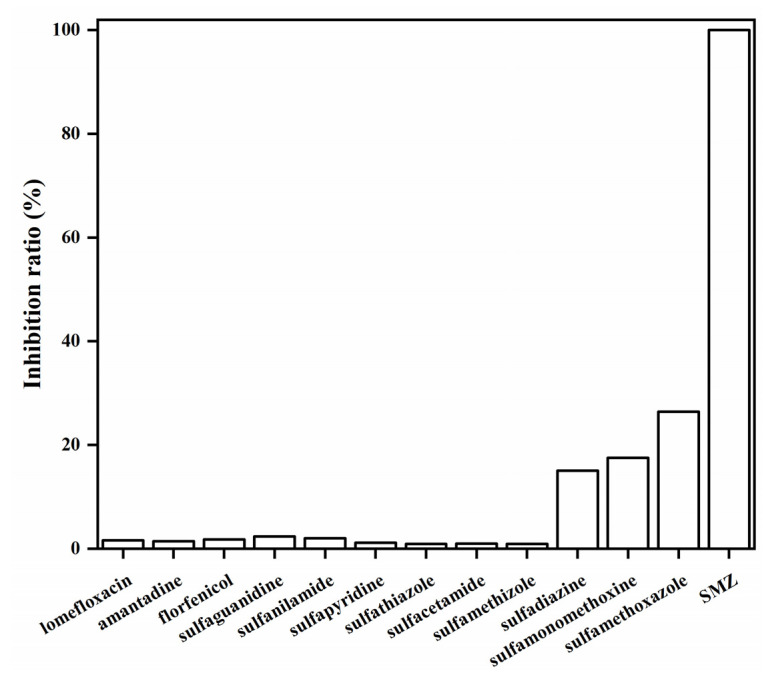
The specificity of QBs-LFIA was tested at a concentration of 20.0 ng/mL with structurally similar analogs.

The specificity of the QBs-LFIA was assessed by running ten other SA drugs and three non-SA antibiotic drugs. As shown in Figure 9, the inhibition ratio of the QBs-LFIA for the SMZ-spiked PBS sample was 100%, while those of sulfadiazine, sulfamonomethoxine, and sulfamethoxazole were 15.5, 17.5, and 26.4%, respectively. In addition, the inhibition ratio was less than 3% in ten other drugs. These results demonstrate that the QBs-LFIA is able to specifically detect SMZ.

##### Recovery Experiment

Under the optimal process parameters, different SMZ concentrations were detected by the QBs-based immunochromatography method. The standard curve was drawn by using the fluorescence intensity of the T line as the ordinate and the logarithm of the corresponding standard concentration as the abscissa. As shown in Figure 8B,C, the fluorescence intensity gradually decreases with the increase of SMZ concentration. The equation of the standard curve is y = −8167 log (x) + 10,788 (R^2^ = 0.9926), y = −5943 log(x) + 8502 (R^2^ = 0.9924) where y represents the fluorescence intensity and x represents the logarithm value of SMZ concentration. By calculation, the LOD of SMZ in these samples detected by QBs-based immunochromatography was 0.1105 ng/mL, 0.0955 ng/mL and the corresponding linear detection range was 0.2–12.5 ng/mL, 0.1–15 ng/mL. The above results indicate that the QBs-LFIA method can realize the high-sensitivity detection of SMZ residue in chicken and milk samples.

The recovery of QBs-LFIA for the determination of 0.05, 0.5, and 5 ng/mL of SMZ in chicken and milk were examined using the test strips. Table 2 shows the average QBs-LFIA recoveries of 80.9–109.4% with CV of 4.88–5.39% in chicken sample and 84–101.6% with CV of 6.48–11.32% in milk sample. These data indicated that the QBs-LFIA could reliably and precisely determine SMZ in chicken and milk samples.

### 3.3. Detection Results of Real Samples

Table 3 showed the detection results of 16 real samples using this method and HPLC. The detection result of the method was in good agreement with that of the HPLC method.

## 4. Conclusions

In this work, we established a rapid and sensitive methods based on QBs-LFIA for the detection of SMZ in chicken and milk samples. The QBs-LFIA had LODs of 0.1138, 0.0955 ng/mL and corresponding linear ranges of 0.2–12.5, 0.1–15 ng/mL in chicken and milk samples, respectively. The recovery experiment and HPLC validation manifested that the method endows with good accuracy and reliability. This type of LFIA have a promising application to promote the comprehensive development of LFIA.

## Figures and Tables

**Table 1:** Comparison of the linear range, LOD, and specificity of this method and others.

Methods	Target	Detection Samples	Linear Range	LOD	Specificity		Reference
					Structure-Relative Compounds	Cross-Reactivity (%)	
Traditional LFIA	SMZ	Meat and egg	-	2 ng/mL	-	-	[23]
MI-ELISA		Swine muscle	100–3200 μg/L	6.8 ng/mL	Sulfapyridine	3.1	[6]
					Sulfadiazine	5.0	
					Sulfamerazine	7.0	
Fluorescence polarization immunoassay (FPIA)		Milk	5–58 ng/mL	1.6 ng/mL	SMZ	7.74	[38]
					Sulfamerazine	61.3	
					Sulfamoxole	415.98	
QBs-LFIA		Chicken	0.2–12.5 ng/mL	0.1138 ng/mL	SMZ	15.5	This work
					Sulfamonometh-oxine	17.5	
		Milk	0.1–15 ng/mL	0.0955 ng/mL	Sulfamethoxaz-ole	26.4	

**Table 2:** Recoveries and CVs of chicken and milk samples spiked with different concentrations of SMZ with QBs–LFIA.

Sample	Spiked SMZ (ng/mL)	Recovery (%) ^a^	Standard Deviation	CV (%)
Chicken	0.5	80.9	3.94	4.88
	5	88.8	4.78	5.39
	7.5	109.4	4.79	4.38
Milk	0.5	84	5.44	6.48
	5	101.6	11.5	11.32
	7.5	96	9.08	9.46

^a^ Recovery = (Detected/Spiked) × 100%.

**Table 3:** Detection of SMZ in real samples using this method and HPLC.

Sample Number		QBs-LFIA (*100)	HPLC	Difference between QBs-LFIA and HPLC (%)
1	Chicken	2.07	228	−9.21
2		1.7	184	−7.6
3		1.15	125	−8
4		0.79	82	−2.56
5		0.37	43.9	−15.3
6		0.157	13.72	14.6
7		ND	ND	
8		ND	ND	
9	Milk	1.64	179.8	−8.4
10		1.12	121.7	−7.4
11		1.18	120.5	−1.7
12		0.54	65	−16.9
13		0.51	60	−15
14		0.31	33	−6
15		ND	ND	
16		ND	ND	

*100: The samples were diluted 100-fold and then dropped into the sample pad of the test strip (the reason is that the linear range of HPLC is 100-fold than that of QBs-LFIA).

## Data Availability

Not applicable.
